# Metabogenic and Nutriceutical Approaches to Address Energy Dysregulation and Skeletal Muscle Wasting in Duchenne Muscular Dystrophy

**DOI:** 10.3390/nu7125498

**Published:** 2015-11-26

**Authors:** Emma Rybalka, Cara A. Timpani, Christos G. Stathis, Alan Hayes, Matthew B. Cooke

**Affiliations:** 1 Centre for Chronic Disease, College of Health & Biomedicine, Victoria University, Melbourne 8001, Australia; cara.timpani@vu.edu.au (C.A.T.); christos.stathis@vu.edu.au (C.G.S.); alan.hayes@vu.edu.au (A.H.); matthew.cooke@vu.edu.au (M.B.C.); 2 Institute of Sport, Exercise & Healthy Living, Victoria University, Melbourne 8001, Australia; 3 Australian Institute of Musculoskeletal Science, Western Health, Melbourne 3021, Australia

**Keywords:** Duchenne Muscular Dystrophy, metabolism, mitochondria, nutriceuticals, dietary supplementation

## Abstract

Duchenne Muscular Dystrophy (DMD) is a fatal genetic muscle wasting disease with no current cure. A prominent, yet poorly treated feature of dystrophic muscle is the dysregulation of energy homeostasis which may be associated with intrinsic defects in key energy systems and promote muscle wasting. As such, supplementative nutriceuticals that target and augment the bioenergetical expansion of the metabolic pathways involved in cellular energy production have been widely investigated for their therapeutic efficacy in the treatment of DMD. We describe the metabolic nuances of dystrophin-deficient skeletal muscle and review the potential of various metabogenic and nutriceutical compounds to ameliorate the pathological and clinical progression of the disease.

## 1. Introduction

Characterised as the most severe and aggressive form of all the muscular dystrophies, Duchenne Muscular Dystrophy (DMD) results from a gene mutation at position 21 on the X chromosome and consequently, absent expression of the cytoskeletal protein dystrophin [1]. The loss of dystrophin expression from skeletal muscle and neuronal tissue in which it is normally present as part of a transmembrane protein complex, induces chronic and progressive skeletal muscle wasting which is fatal in all cases. The etiology of the disease is intimately linked to the cytostructural role of dystrophin in providing stability to the sarcolemma, particularly during contraction; regulating the proper expression of components of the sarcolemmal Dystrophin Protein Complex (DPC); and, consequently, maintaining appropriate homeostatic transmembrane ion gradients and cell signaling functionality [2,3,4]. It is widely reported in the literature that the secondary molecular mechanisms ultimately leading to muscle degradation include abnormal calcium (Ca^2+^) homeostasis [5,6]; Ca^2+^-induced necrosis [7]; mitochondrial dysfunction and cellular energy perturbations [8,9,10,11,12]; and satellite cell (stem cells that repair damaged skeletal muscle) exhaustion [13,14]. As skeletal muscle regeneration fails to match degeneration rates and inflammatory activity persists, skeletal muscle becomes infiltrated with fat and connective tissue which limits function and leads to the loss of ambulation in the teenage years [15,16]; ultimately fibrosis of the diaphragm and heart ensues causing respiratory dysfunction, cardiomyopathy and death by the third decade of life [17,18].

Impaired energy metabolism has been historically, and again more recently, regarded in the literature as a key player in the degeneration of dystrophin-deficient muscle (for review see [19]). While a strong rationale exists in support of this bioenergetical dysregulation being a fundamental consequence of increased demand for ATP-dependent intracellular Ca^2+^ buffering and satellite cell-mediated muscle repair, it has alternatively been suggested that metabolic impairments may be linked to the DMD genotype [11,20]. Various metabolic defects have been reported in dystrophin-deficient skeletal muscle, affecting glycolysis, fatty acid metabolism, the tricarboxylic acid (TCA) cycle, mitochondrial electron transport chain and the purine nucleotide cycle (PNC) [21,22,23,24,25,26,27]. Moreover, our group, along with others [8,10,28], has recently demonstrated an impaired capacity for dystrophin-deficient muscle to produce ATP [12]. Collectively, these deficits amount to a 50% reduction in ATP content within dystrophin-deficient myofibres from human patients and animal models [8].

Gene and stem cell therapies represent the only potential cures for DMD, yet, some 20 years following identification of the genetic defect, appropriate curative therapies are not a mainstream treatment [29,30,31]. Currently, DMD patients are treated somewhat successfully with the corticosteroid prednisolone, which while providing therapeutic value to the majority of sufferers, is not without side effects [32]. Thus, treatment protocols that reduce the severity and progression of muscle wasting must continue to be rigorously researched. Since dystrophin-deficient skeletal muscle is underscored by a reduced capacity for energy biosynthesis, and this leaves muscle fibres ill-equipped to buffer the pathophysiological cascade induced by the absence of dystrophin expression, targeting the energy-producing pathways with therapeutic intervention seems logical. Indeed, a solid body of literature has investigated the therapeutic potential of energy-promoting nutriceutical and metabogenic supplements for the treatment of DMD. These studies are reviewed here in.

## 2. Dysregulation of Energy and Protein Metabolism in DMD

Both historically and more recently, DMD has been regarded as a disease of impaired myofibre energy status, with resting ATP levels being half that of healthy control muscle [8,28] and the ATP production capacity of dystrophic mitochondria demonstrably impaired [9,10,11,12]. These deficits are evident in skeletal muscle from human DMD patients as well as from the genetically homologous *mdx* mouse model of the disease [33,34]. Adequate production of ATP is essential to maintain the integrity and function of all cells, and as such, it is synthesized immediately via the creatine phosphagen system, and continuously via the metabolism of glucose via glycolysis and fatty acids via β-oxidation. Generated pyruvate (glucose metabolism) and acetyl CoA (fat metabolism) are subsequently shuttled into the linked TCA cycle and electron transport chain in the mitochondria and ATP is generated via the process of oxidative phosphorylation (OXPHOS). Besides the direct uptake of glucose by the glucose transporter (GLUT), glucose can be metabolically extracted from stored glycogen, and/or synthesized from pyruvate, amino acids (AAs) and lactate when blood glucose levels deplete. Skeletal muscle tissue also relies heavily on alternative pathways for ATP production in that of the cytoplasmic pentose phosphate pathway (PPP).

**Figure 1:**
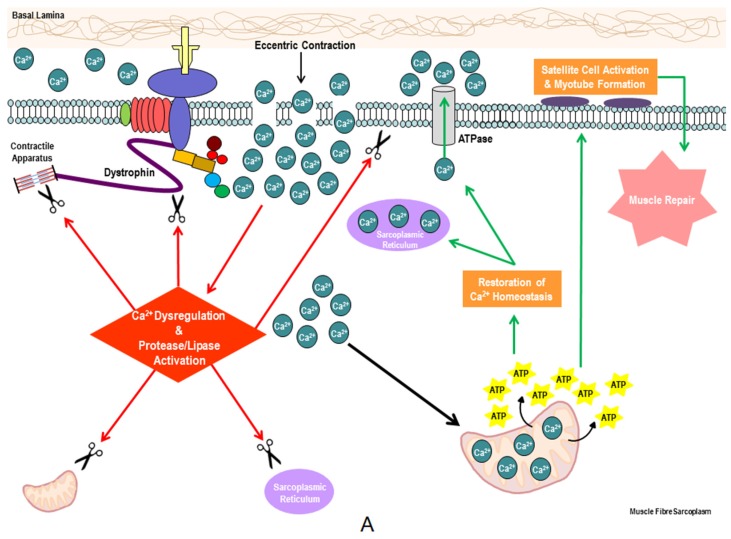

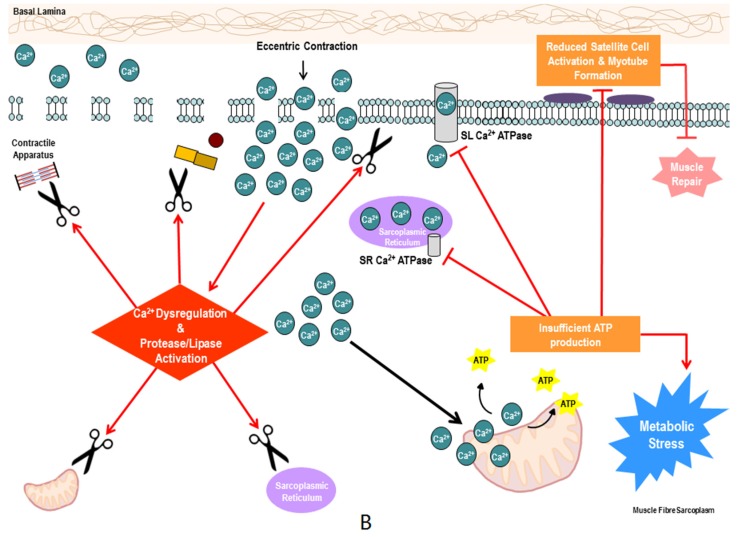
The role of ATP in the mitigation and recovery of eccentrically-induced damage in (**A**) healthy and (**B**) dystrophin-deficient skeletal muscle fibres. Eccentric damage of healthy muscle (**A**) potentiates Ca^2+^ influx from the extracellular space and increases the intracellular Ca^2+^ concentration. Proteases and lipases activated by Ca^2+^, cause damage to the contractile apparatus, mitochondria, sarcoplasmic reticulum and the muscle membrane. In healthy muscle, Ca^2+^ uptake into the mitochondria stimulates oxidative phosphorylation and ATP production is increased to support ATP-fuelled Ca^2+^ extrusion pumps in the muscle membrane, sarcoplasmic reticulum and mitochondria, thus restoring intracellular Ca^2+^ homeostasis and mitigating the severity of damage. ATP also fuels satellite cell replication and skeletal muscle repair, which is activated by the inflammatory response. In dystrophin-deficient skeletal muscle (Figure 1B), the increased propensity for membrane rupture during eccentric contraction causes the same, albeit amplified, degenerative cascade. Teamed with mitochondrial dysfunction, however, ATP production is insufficient to fuel the ATP-dependent buffering of Ca^2+^ influx to mitigate damage—degenerative activity is therefore amplified. There is also a limited capacity for skeletal muscle repair due to the energy-demanding nature of cell proliferation. The consequence is metabolic stress, progressive muscle degeneration, insufficient repair of degeneration and muscle wasting.

Our group has previously shown a reduced propensity for NADH redox (Complex I)—compared to FADH_2_ redox (Complex II)-stimulated OXPHOS in *mdx* mitochondria [12]—albeit both states were depressed compared to healthy control mitochondria. This suggests in the first instance that *mdx* mitochondria display a reduced capacity for pyruvate flux-mediated OXPHOS, which likely reflects an inherent reduced capacity for glycolytic flux *in vivo*. Our data is consistent with several studies that have demonstrated a reduced capacity for glycolysis [21,22,23,24,25] and reduced expression of the cellular glucose uptake and glycolytic flux regulator, neuronal nitric oxide synthase (nNOS). nNOS normally co-localises with dystrophin at the sub-sarcolemma [35,36]. In the absence of dystrophin expression, nNOS becomes a target of hyper-activated calpains leading to reduced expression and nNOS-mediated NO production. As nNOS and/or nNOS-generated NO are strong regulators of glucose uptake and flux through the glycolytic enzyme cascade (particularly during muscle contraction) [37,38], it is likely that reduced substrate availability is a precursor to energy system de-regulation. However, defects in fat oxidation have also been reported [39,40,41,42,43,44,45], which in conjunction with our data, suggests a fundamental defect at the mitochondrial level that induces deregulation of all metabolic systems. It is the working hypothesis of our group, that mitochondrial pathology forms the basis of DMD aetiology alongside dystrophin-deficiency [19] (Figure 1), such that much like the damage following eccentric muscle injury (Figure 1A), dystrophin-deficiency-mediated damage could be mitigated if ATP availability was sufficient. Teamed with mitochondrial pathology, however, a “two-hit” scenario exists whereby a reduced capacity for ATP synthesis and perturbations in myofibre energy homeostasis (*i.e.*, metabolic stress) exacerbates muscle degeneration, reduces regenerative capacity and ultimately, promotes muscle wasting (Figure 1B). During metabolic stress, a cell signaling cascade is initiated in skeletal muscle which inhibits protein synthesis and promotes muscle catabolism via autophagy. As such, ATP utilization is spared and AAs stored as skeletal muscle tissue are made available to metabolism to increase ATP synthesis and restore energy homeostasis [46]. This is achieved predominantly through the activation of adenosine monosphosphate (AMP)-activated protein kinase (AMPK) which is phosphorylated by rising AMP levels. It has been established that both increased AMPK and autophagic activity are a feature of dystrophin-deficient skeletal muscle [47]. As such, a proportion of the muscular hypercatabolism observed in dystrophin-deficient muscle may be attributable to autophagy. Consistent with this, Rennie *et al.* [48] demonstrated a 2.5-fold increase in protein turnover and a 6.5-fold reduction in capacity for muscle-specific protein synthesis in DMD patients that coincided with a 63% increase in leucine oxidation. Okada *et al.* [49] also report higher urinary 3-methylhistidine excretion as an indicator of increased protein turnover in DMD patients, and suggest that the maintenance requirement of dietary protein intake is 70% higher in DMD patients compared to healthy controls. Both metabolic stress and insufficient protein/AA intake are likely contributors to an overall reduced capacity for muscle protein synthesis and the maintenance of muscle mass in hypercatabolic dystrophin-deficient skeletal muscle. Indeed, AMPK activation is a strong inhibitor of protein synthesis [50], while increased plasma AA concentration is a strong stimulator of it [51]. Thus, targeting metabolic and protein synthesis pathways as therapeutic intervention points is a promising notion.

## 3. Therapeutic Potential of Metabogenic and Nutriceutical Supplements

### 3.1. Amino Acids and Protein Isolates

In addition to their role as building blocks for protein synthesis, AAs directly orchestrate a cell-signaling cascade that stimulates intracellular protein synthesis—especially in the case of the essential AAs [52,53,54]. This occurs via activation of the phosphatidylinositol (PI) 3-kinase-mammalian target of rapamycin (mTOR) signaling pathway in which AAs demonstrably act on various target molecules to elicit both activating and inhibitory effects on mRNA translation and cellular hypertrophy [55,56,57]. AAs stored in muscle protein are also important energy reservoirs and act as substrates for gluconeogenesis and TCA cycle intermediates to supplement energy metabolism during periods of substrate deprivation (*i.e.*, starvation) and/or metabolic stress [58,59,60]. Several AA-like molecules are also crucial to skeletal muscle maintenance and function, such as creatine (Cr)—which functions importantly as a high-energy storage molecule and stimulant of mitochondrial ATP production—and taurine.

#### 3.1.1. Creatine

Increasing intramuscular phosphocreatine (PCr) levels via oral Cr loading has been well documented [61,62,63] and has become a mainstay of athletes seeking putative performance enhancement—especially with respect to strength, speed and power. Various physiological benefits have been attributed to increases in the total creatine (TCr = PCr + Cr) status of muscle, including: (1) enhanced mitochondrial ATP flux via the PCr shuttle [64,65,66,67]; (2) stimulation of glycolysis [68,69]; (3) improved buffering of both the ATP:ADP ratio via the phosphagen system [70,71,72] and intramuscular pH [73]; and (4) enhanced protein synthesis (particularly when accompanied with resistance exercise) [74]. As such, several studies have examined the efficacy of Cr supplementation as a therapeutic intervention in both human DMD patients [75,76,77,78,79] and in the *mdx* mouse model of DMD [9,80,81].

In a randomized double-blind cross-over study, Louis *et al.* [78] examined the therapeutic efficacy of a 3 g·day^−1^ Cr supplementation protocol for 3 months in DMD patients and demonstrated benefits in measures of muscle performance—specifically in doubling the time to fatigue at 75% maximum voluntary contraction (MVC) and allaying the increase in total joint stiffness that was observed in untreated controls over the 3-month trial. The authors did not observe increases in lean body mass, reduction in serum CK levels or changes in creatinine excretion rate, suggesting that Cr may provide alternative benefits aside from enhancing cellular energetics and preventing muscle damage [78]. In a similar study with 30 DMD boys (50% of whom also maintained corticosteroid treatment throughout), Tarnopolsky *et al.* [77] supplemented Cr at a dosage rate of 0.1 g·kg^−1^·day^−1^ for 4 months (followed by a 6 week washout phase and 4 months of placebo) and also reported improvements in dominant hand-grip strength and the maintenance of strength over time, albeit no improvement in functional task decline or measures of pulmonary function. A particularly interesting feature of Tarnopolsky’s study was the finding of significantly increased fat-free mass after a four-month supplementation protocol [77]. This suggests that in this trial, Cr supplementation either promoted net muscle accretion or reduced the rate of muscle degradation induced by the disease pathology. Cr supplementation, however, had no effect on body fat percentage [77]. 

More recently, Banerjee *et al.* [82] investigated the effect of Cr supplementation on skeletal muscle bioenergetics. In a randomized, placebo-controlled (500 mg vitamin C) single blinded experiment, steroid-naïve DMD boys were supplemented orally with Cr at a dosage rate of 5 g·day^−1^ for 8 weeks. The study reported an increase in the PCr/P_i_ ratio, PCr content and ATP peaks (^31^P MRS) that translated at a functional level, to increased strength in Cr-treated DMD patients [82]. This highlights that Cr supplementation augments functional improvements by positively regulating skeletal muscle energy homeostasis.

Beneficial effects have also been observed consistently in *mdx* skeletal muscle. Louis *et al.* [81] supplemented *mdx* mice with Cr in both the food (47 g·kg^−1^) and water (14 g·L^−1^) supply for 30 days and reported a 12% increase in Extensor Digitorum Longus (EDL) TCr content that effectively restored normal resting levels (as depicted by controls). This corresponded histologically with a reduction in pseudohypertrophic muscle mass and mean fibre surface area. In this study, Cr supplementation had no protective effect on susceptibility to stretch-induced fibre injury or the accumulation of centrally nucleated (regenerative) fibres in adult EDL muscle. Cr supplementation also induced a significant increase in half relaxation time (½ RT) in both *mdx* and normal mice and a highly significant increase in the total Ca^2+^ content of the gastrocnemius muscle that was not observed in Cr-supplemented normal mice [81]. This data suggests that Cr-supplementation failed to allay the dystrophic phenotype. 

Pulido *et al.* [80] has published the only study to date investigating the effect of Cr supplementation on intracellular Ca^2+^ handling in dystrophic muscle. Cultured *mdx* myotubes were supplemented *in vitro* with 30 mM Cr at the onset of myocyte fusion and cytoplasmic Ca^2+^ was quantified using the Ca^2+^-specific fluorophore Fura-2. Cr was shown to significantly inhibit dystrophy-induced sarcoplasmic elevations in Ca^2+^ concentration after several days of supplementation. This was attributed to enhanced SR Ca^2+^ ATPase activity, albeit direct measurement of SR Ca^2+^ was not made and thus improvements in sarcolemmal and mitochondrial ATPase activity could not be ruled out. Ca^2+^ influx rates remained unaffected between Cr-supplemented and unsupplemented groups, indicating definite improvements in Ca^2+^ buffering capacity rather than a reduction in entry [80]. This study most importantly demonstrated that Cr-induced improvements in sarcoplasmic Ca^2+^ buffering and improved myotube survival rates [80]. An extension of this research by Passaquin *et al.* [83] supported Pulido’s findings, demonstrating delayed onset and reduced severity of initial degenerative cycles in young mice, which was accompanied by enhanced mitochondrial oxidative function. Whilst the findings of Pulido *et al.* [80] and Passaquin *et al.* [83] differ from those of Louis *et al.* [81] with respect to the effects of Cr supplementation on skeletal muscle Ca^2+^ handling, it is possible that the absolute gastrocnemius Ca^2+^ concentration ascertained by Louis *et al.* [81] is misrepresentative of the fact that Cr supplementation might increase cell survival duration by increasing the buffering of Ca^2+^ from the sarcoplasm into subcellular compartments and retaining it within. Thus whilst a greater net Ca^2+^ concentration of myofibres would be observed, relative sarcoplasmic Ca^2+^ concentration would be significantly decreased by the better Ca^2+^ buffering capacity afforded by Cr. That CK is demonstrably linked to the sarco(endo)plasmic reticulum Ca^2+^ ATPase (SERCA) indeed indicates that improved uptake into the SR is likely [84,85], and thus that maintaining energy homeostasis in fundamental to moderating the pathological damage of dystrophin-deficient skeletal muscle.

Taken together, the collective data suggests that Cr supplementation has the ability to offset the degenerative features of dystrophin-deficient muscle by improving PCr content, ATP rephosphorylative capacity, mitochondrial function and Ca^2+^ compartmentalization within skeletal muscle to reduce/offset damage. While the hallmark clinical signs of muscle degeneration (such as serum CK and urinary creatinine excretion) are still evident in patients following Cr supplementation, the functional improvements in strength and fatigability observed suggest that supplementative Cr is still a valuable therapeutic adjunct to improve patient quality of life.

#### 3.1.2. Glutamine

Glutamine is the most abundant free AA in the plasma and skeletal muscle. Glutamine has many physiological roles within the body, but its effect on protein turnover is the most pertinent in relation to DMD. Since glutamine is predominantly produced by the muscle, dystrophin-deficiency-induced muscle degradation leads to a decrease in glutamine turnover and low intramuscular glutamine concentration [86]. This results in a negative whole-body leucine balance [86]. With this in mind, the need for extra glutamine for protein metabolism support may be increased in DMD patients. The first study to demonstrate glutamine’s potential effect on whole body protein balance was by Hankard *et al.* [87]. The study’s primary aim was to examine the acute anabolic effects of glutamine ingestion on 6 young boys with DMD. Despite no effect on non-oxidative leucine disposal (a marker of protein synthesis), oral glutamine supplementation (800 µmol·kg^−1^·L^−1^) decreased leucine and glutamine release from protein degradation, in addition to glutamine *de novo* synthesis, suggesting a possible protein-sparing effect in DMD children [87].

To confirm these initial findings, but also determine if a longer supplementation could be more beneficial, Mok *et al.* [88] randomised 26 DMD boys to ingest either glutamine (0.5 g·kg^−1^·day^−1^) or an iso-nitrogenous, nonspecific AA mixture (0.8 g·kg^−1^·day^−1^) for 10 days. The rate of leucine appearance and endogenous glutamine due to protein breakdown (estimates of whole-body protein degradation) were decreased in both groups following the 10 day supplementation period. Interestingly, plasma glutamine and AA concentrations did not increase following the supplementation period and the authors suggest that glutamine’s effect on whole-body protein metabolism may not be solely driven by substrate availability. Alternatively, glutamine uptake by the intestine might be sparing other AAs and substrates from degradation, and thus could be driving the protein-sparing effect in DMD [88]. To explain the molecular basis for glutamine’s anti-proteolytic effect in DMD children, the same author and lab used animal models to demonstrate a reduction in GSSG/GSH (the major regulator of the cellular redox potential and a widely used indicator of oxidative stress) and ERK 1/2 activation (a signaling kinase involved in muscle breakdown) in dystrophic skeletal muscle of young *mdx* mice following daily intraperitoneal injections of L-Gln (500 mg·kg^−1^·day^−1^) or 0.9% NaCl for 3 days [89].

While these results are promising, it is important to determine if glutamine’s protein-sparing effects could translate into functional benefits, but more importantly, clinically relevant benefits. In a randomised controlled trial (RCT), ambulant DMD boys (*n* = 30) ingested both glutamine (0.5 g·kg^−1^·day^−1^) and placebo (maltodextrin) in a cross-over design for a period of 4 months [90]. The study showed no improvements in walking speed and other functional tests, body composition and markers of myofibrillar protein breakdown following the supplementation period. Despite some limitations in the study (variability in functional measures, response to placebo in children in RCTs *versus* adults), the routine use of glutamine in this population could not be supported. This was also confirmed in another RCT trial that tested the efficacy of glutamine in DMD boys aged 4–10 years [91] using a parallel trial to compare 6 months of oral glutamine (0.6 g·kg^−1^·day^−1^) (*n* = 19) with placebo (*n* = 16). This study showed no significant effects on manual muscle testing scores of 34 muscle groups, or on quantitative measures of muscle strength of bilateral elbow flexors and extensors, knee flexors and extensors and grip. Therefore, despite previous experimental data in *mdx* mice [89,92] and short term clinical studies showing improvements in protein metabolism and whole body strength, especially in younger dystrophic males [87,88]; current evidence in RCTs is not convincing and clearly warrants further investigation.

#### 3.1.3. Taurine

Taurine is another free AA-like molecule that is abundant in skeletal muscle in addition to several other highly metabolic tissues including brain and liver. Though the majority of taurine formation occurs in the liver, other cells are also able to synthesise taurine from cysteine, including kidney, brain and to a lesser extent, skeletal muscle [93]. Taurine exerts a wide spectrum of actions including fluid concentration control and antioxidant and anti-inflammatory effects. In skeletal muscle, taurine plays an integral role in ion channel function and Ca^2+^ homeostasis [94,95,96,97]. Similar to intramuscular glutamine, dystrophic muscles seem to be deficient in taurine, which is likely a consequence of excess cysteine disposal [98]. Normalising taurine levels in dystrophic muscles may help to stabilize membrane integrity and reduce inflammation and oxidative stress, and therefore decrease muscle dystrophinopathy. Previous studies have demonstrated that treatment of dystrophic muscles with taurine (10%, w/w in chow) is relatively safe, but importantly, can lead to improvements in grip strength, prevention of exercise-induced muscle weakness and restoration of Ca^2+^ homeostasis [99,100]. In a study designed primarily to determine the potential of nuclear magnetic resonance spectroscopy as a tool to monitor histology, researchers showed that in muscles of *mdx* mice treated with glucocorticoid, taurine levels were increased [101]. This could suggest that taurine, in part, may be contributing to the improved pathology normally associated with glucocorticoid treatment [101]. Recently, intramuscular taurine deficiency in *mdx* mice was ameliorated by l-2-oxothiazolidine-4-carboxylate (OTC), a cysteine precursor, administration [98]. Mice received 0.5% (w/v) OTC in their drinking water for a period of 6 weeks (from 6 to 12 weeks of age) which equates to approximately 100 µM of cysteine per day. Six weeks of OTC treatment increased taurine content in the liver (similar extent as controls) and plasma of *mdx* mice which resulted in a decrease in protein thiol oxidation (oxidative stress), decreased pathology and increased forelimb grip strength. 

Our own work has shown that 4 weeks of dietary taurine supplementation (3% w/v in drinking water) increased taurine content in *mdx* mice [102]. This resulted in enhanced fatigue resistance and better recovery of force development of muscles contracted *ex vivo* [102]. Given no alternations in calcium-handling proteins were observed, it is possible that improvements in metabolic function contributed to the effects observed. Clearly further experimental work is needed to establish whether these benefits in dystrophic animal models of DMD can be translated into human DMD patients, but also to understand the precise cause of taurine deficiency and determine which taurine-enhancing intervention is best able to afford functional and clinically significant benefit to DMD patients.

#### 3.1.4. Arginine

As previously mentioned, the lack of dystrophin in skeletal muscle disrupts the recruitment of nNOS to the sarcolemma which negatively affects endogenous skeletal muscle NO production. NO is involved in many physiological roles including force production (excitation-contraction coupling), blood flow autoregulation, myocyte differentiation, respiration, and glucose homeostasis [38]. Increasing NO can be achieved via administration of l-arginine (the substrate for nNOS). Using this method, 4 month old female *mdx* mice were given l-arginine treatment (200 mg·kg^−1^, intraperitoneal injection) for 5 out of 7 days for 6 weeks. l-arginine treatment upregulated utrophin (another cytoskeletal protein with over 80% homology with dystrophin) expression 2- to 3-fold in dystrophic *mdx* muscles and lead to a 35% reduction in necrotic zones [103]. Additionally, when l-arginine (0.375% in drinking water) is given in combination with Deflazacort (a glucocorticoid) for 3 weeks, quadriceps muscles were spared from injury-induced regeneration, despite an increase in membrane permeability following exercise [104]. Performance was also improved with an increase in the distance (km) run voluntarily by individual mice. Although early results suggest a potential new treatment for improving the quality of life for boys with DMD, similar to taurine, further clinical trials need to be performed. This is especially true, as nNOS content is notably reduced to critically low levels in the skeletal muscle of DMD patients [35,36], but variably, if at all, in *mdx* mouse skeletal muscle. As l-arginine is a substrate for nNOS-mediated NO production, a lack of nNOS protein would theoretically render supplementative therapy useless at increasing skeletal muscle NO levels. Moreover, since l-arginine can have adverse side effects, other methods to increase NO, might be preferable for NO-based therapy in muscular dystrophy [105].

Indeed a series of recent studies have demonstrated the efficacy of NO-donating non-steroidal anti-inflammatory drugs (NSAIDS) in reducing muscle wasting and promoting regenerative capacity in the *mdx* mouse model and in human DMD patients [106,107,108,109]. However, due to the extremely short half-life of NO, there is a questionable capacity for highly reactive NO donors to mediate effects outside of the vasculature. It is probable, therefore, that the beneficial effect afforded by NO-donor-NSAID therapies is more likely related to a modulation of blood flow/systemic O_2_ delivery and/or inflammatory/immunological pathways, and, therefore, skeletal muscle regenerative capacity; rather than NO-mediated effects within the skeletal muscle itself. Like NO-donor therapy, pharmacologically amplifying/extending the NO-mediated-cGMP signal using the phosphodiesterase 5 (PDE5) inhibitors sildenafil (Viagra^®^) and tadalafil (Cialis^®^) could be useful for circumventing the poor endogenous NO production caused by reduced nNOS content to afford therapeutic efficacy in dystrophin-deficient skeletal muscle. In *mdx* mice, Percival *et al.* [110] demonstrated that 14 weeks of sildenafil treatment significantly reduced damage to, and fibrosis of, diaphragmatic skeletal muscle fibres. However, in a later study, the same group concluded that therapeutic efficacy is not via metabogenic effects on the mitochondria as sildenafil treatment actually reduced the ATP content of hind limb skeletal muscle. The body of literature since suggests that the benefits of pharmacological NO-cGMP signal amplification lie at the vascular level, and in increasing blood flow and O_2_ delivery to skeletal and cardiac muscle particularly during exercise [111,112]. Most interesting, is a recent randomized, double-blind, placebo-controlled, cross-over design clinical study in BMD patients, which demonstrated no effect of sildenafil on exercise-induced blood flow, functional tests, or skeletal muscle oxidative capacity; but rather concomitant deficiency of PDE5 receptor expression alongside nNOS deficiency in dystrophin-deficient skeletal muscle [113]. This suggests that the propensity for pharmacological inhibition of PDE5 as a therapeutic target to enhance the NO-cGMP axis is diminished in human patients in particular. Thus alternative avenues for enhancing NO production and downstream signaling pathways would be useful. Nutritional supplementation of dietary nitrate has been consistently shown in the literature to increase the total nitrate/nitrite/NO pool in skeletal muscle and generate NO independent of nNOS [114,115]. It may, therefore be of therapeutic value to dystrophin-deficient skeletal muscle as an alternative means of restoring endogenous NO production within skeletal muscle, as has been suggested in diabetic skeletal muscle [116,117].

#### 3.1.5. Whey Protein Isolates

An extract of soluble protein fractions from bovine milk, whey protein (WP) supplements are popular as an established method of enhancing muscle anabolism and hence athletic performance [118,119,120]. It has been suggested that WP offers considerable benefits over other high quality protein sources by constituting a higher concentration of essential AAs (45–55 mg·100 g^−1^) [121], and thus conveying a higher biological value—descriptive of its ability to provide and retain nitrogen in a balanced interplay of essential and non-essential AAs [122]. In comparison to the other high quality dairy protein, casein, WP also claims a higher protein efficiency ratio (PER) of 2.6 *versus* 3.2, thus eliciting a larger rate of weight gain per gram of protein consumed over time [122]. Indeed, the unique physical properties of WP in the gastrointestinal system are likely to convey its benefits—unlike casein that clots in the stomach resulting in slowed release into the small intestine, and thus significant hydrolysis prior to absorption, WP immediately enters the small intestine in which its progressive hydrolysis is relatively slow, providing a unique delivery of both AAs and peptides [123,124]. Because of this difference, WP consumption elicits an acute peak in serum AA concentration [124,125,126] that when accompanied with mixed macronutrients, demonstrably induces increased rates of muscle protein synthesis and net gains in systemic protein deposition [126]. WP may also offer various other benefits specific to its high concentration of both the sulphur-containing AA cysteine for its immunological and anti-oxidant modulatory role [127], and branched-chain AA leucine for its skeletal muscle protein regulating role [128,129].

**Figure 2:**
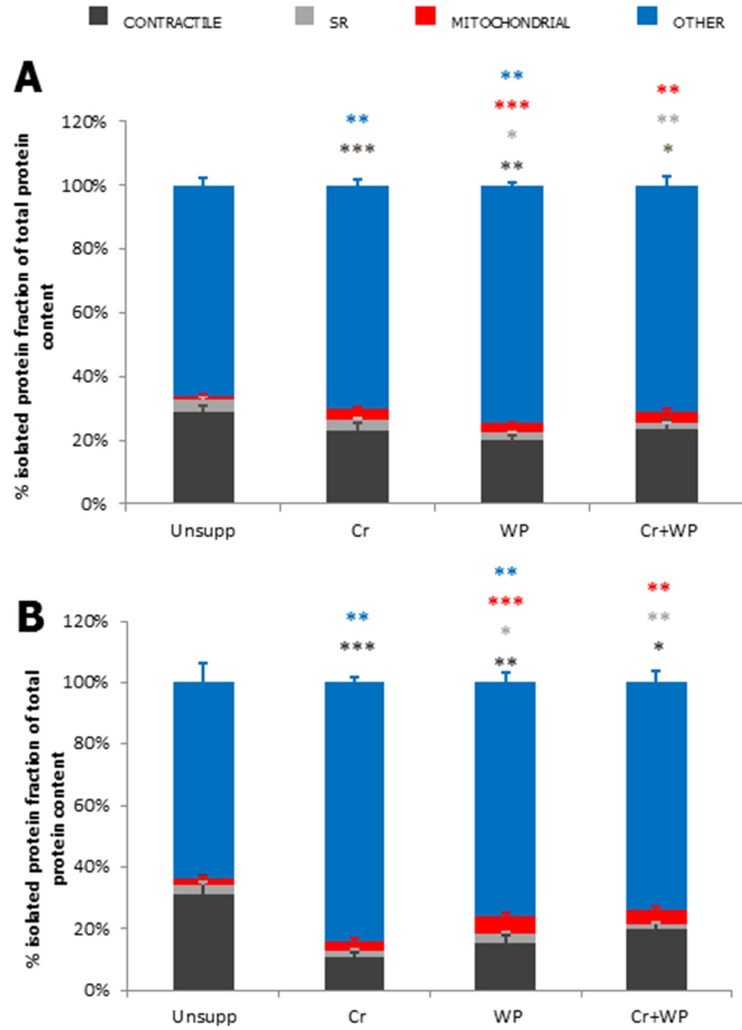
Subfractional protein distribution following dietary Cr and WP supplementation in gastrocnemius (**A**) and diaphragm (**B**) from the *mdx* mouse model of DMD. Contractile proteins are indicated by dark grey bars, sarcoplasmic reticular (SR) proteins are indicated by light grey bars, mitochondrial proteins are indicated by red bars and the remaining protein pool (containing structural and nuclear proteins) is highlighted by the blue bars. Data is expressed as the mean % subcellular protein fraction of the total protein pool ± SEM. Significance was considered as *p* < 0.05 compared to unsupplemented *mdx* control muscle and is indicated by asterisk whereby: * *p* < 0.05, ** *p* < 0.01, and *** *p* < 0.001 all different from Unsupp; *n* = 8–10. Asterisk colour denotes the corresponding subcellular fraction as depicted in the key, and above.

Whilst there is no published data to date describing the effect of WP (or casein/other non-dairy protein isolates) supplementation on skeletal muscle preservation in DMD patients, it is clear from isolated AA supplementation trials (described previously) that increasing systemic AA status could be of therapeutic benefit, particularly if the aforementioned hypothesis of dystrophinopathy-induced AA deficit is true. We have preliminary data demonstrating that dietary supplementation of *mdx* mice with WP (16% in chow), both alone and in combination with Cr monohydrate (1% in chow) (WP + Cr) for 6 weeks, increases the total protein content of the gastrocnemius and diaphragm ([130]; Figure 2). However, most interestingly, WP and WP + Cr supplementation significantly decreases the contractile protein fraction of the total protein pool while inducing increases in the mitochondrial and structural protein fraction in both gastrocnemius and diaphragm (Figure 2). Our preliminary data suggests that in times of metabolic stress when mTOR signaling is inhibited by AMPK and downstream activators drive mitochondrial biogenesis, AA uptake into cells is directed toward mitochondrial fission, growth and function. Indeed, our data in healthy trained humans also suggests an augmentative effect of WP on contractile protein accretion-independent force production [131]. While Cr supplementation demonstrably induced greater increases in fibre size, contractile protein content and strength gains, the force production gains observed following Cr + WP supplementation were independent of fibre size [131]. We speculate that WP modulates contractile function via direct effects on the sarcoplasmic reticular calcium-handling apparatus and/or mitochondrial ATP production, albeit we are yet to confirm this in healthy humans or DMD patients/animal models. Considerable research would thus be required to establish WP supplementation as a therapeutic adjunct for the treatment of DMD.

### 3.2. Mitochondrial Co-Factors and Modulators

#### 3.2.1. Coenzyme Q10 (CoQ10)

CoQ10 is an essential cofactor found naturally in practically every cell in the human body. CoQ10 is lipid-soluble and primarily acts as an electron carrier in the mitochondrial respiratory chain. CoQ10 is also a strong antioxidant which is able to reduce oxidative radicals and modulate the mitochondrial permeability transition pore (MPTP) which could potentially be of benefit in DMD muscle to decrease mitochondrial Ca^2+^ accumulation, swelling, dysfunction and mitochondrially-regulated apoptosis [132]. Previous oral forms of CoQ10 have had limited success at increasing mitochondrial CoQ10 content due to solubility issues, although recent advances in delivery (*i.e.*, fast melt formations) have improved mitochondrial uptake and as a consequence, increased performance [133]. Tissue deficiencies or suboptimal serum levels of CoQ10 have been reported in a wide range of medical conditions, including cardiomyopathies and degenerative muscular and neuronal diseases [134,135,136]. Given DMD patients often develop heart problems as the disease progresses, but are also characterised by skeletal muscle weakness due to excessive muscle damage, one potential additional therapy in DMD could be CoQ10 supplementation.

Early studies in dystrophic mice have shown promising results with increases in both physical performance and survival following CoQ10 administration [137,138]. In human patients, one of the earliest studies examined CoQ10 administration for a minimum of 40 weeks to an infant (1–2 years age) and young boy (3–5 years) with preclinical DMD and reported a significant reduction in creatine kinase, an indirect maker of muscle damage [139]. The first double-blind trial was conducted with twelve patients, ranging from 7 to 69 years of age, having progressive muscular dystrophies and neurogenic atrophies including Duchenne, Becker, and limb-girdle dystrophies, myotonic dystrophy, Charcot-Marie-Tooth disease, and Welander disease. Baseline CoQ10 blood levels were low and ranged from 0.5 to 0.84 µg·mL^−1^. Patients were treated for three months with 100 mg daily of CoQ10 and a matching placebo. Following the supplementation period, improved physical well-being was observed for half of the CoQ10-treated patients but for none of the placebo patients. Following crossover, 75% of the initial placebo patients showed improvement with CoQ10-treatment and five of the six patient who were initially treated with CoQ10 maintained improved cardiac function in the placebo phase. Only one of the six patients who crossed over from initial CoQ10 to placebo treatment relapsed. The authors suggested that while effective for the majority of DMD patients, a dosage of 100 mg was potentially too low, albeit effective and safe [140,141].

Recently, a pilot trial was conducted to determine whether the addition of CoQ10 to a stable steroid regimen could further preserve or increase muscle strength in DMD [142]. Participants underwent 3 months of lead-in evaluation at their pre-established dose of prednisone, and then began a CoQ10 dose-finding period at a starting dose of 90 mg CoQ10 daily and a dose escalation of 30 mg daily until achieving a serum CoQ10 level of 2.5 μg·mL^−1^. Following the treatment of an initial 3 patients and failure in achieving desired levels, the initial dose was increased to 400 mg with dose escalations of 100 mg and delivered in conjunction with a small fatty snack (ice cream). Once the minimum level was achieved, participants began the 6 month evaluation period, with repeat drug level monitoring at each visit. Dose adjustments were performed as needed, and reductions were only performed if there were side effects considered to be related to the drug. The study showed that the addition of CoQ10 to prednisone treatment in DMD improved muscle strength (8.5%) above and beyond steroid-related improvements. These limited results suggest further testing of CoQ10 as an additional therapy in DMD is warranted.

A synthetic analogue of coenzyme Q that is currently employed in the treatment of vascular and degenerative diseases of the central nervous system is Idebenone (2,3-dimethoxy-5-methyl-6-(10-hydroxydecyl)-1,4-benzoquinone). Idebenone is a short-chain benzoquinone with strong antioxidant properties and capacity to improve mitochondrial respiratory chain function and cellular energy production [143]. Limited studies have examined the therapeutic benefit of Idebenone in dystrophin-deficient muscular dystrophy. An early preclinical trial examined long term (age 4 weeks until 10 months) administration in the *mdx* mouse [144]. In this observer-blinded placebo-controlled *in vivo* study, Idebenone treatment was demonstrably cardioprotective and improved voluntary running performance. Based on the preclinical evidence, a recent phase IIa randomized controlled clinical trial was conducted to investigate the efficacy and tolerability of Idebenone in children with DMD, focusing on cardiac and respiratory endpoints [145]. Twenty-one 8–16 year old DMD patients received either oral Idebenone (150 mg) or matching placebo, three times daily (during meals) for 52 weeks. Idebenone was safe and well tolerated and treatment compliance was excellent. Compared to patients on placebo, patients receiving Idebenone showed a trend for improvement of peak systolic radial strain in the left ventricular inferolateral wall, the region of the heart that is earliest and most severely affected in DMD. Parameters for respiratory muscle weakness (Peak expiratory flow (PEF), maximum inspiratory pressure (MIP)) deteriorated in the placebo group but improved in the Idebenone group, with a statistically significant treatment effect of Idebenone on PEF. An important limitation of this study was the relative small number of patients, which in combination with the fixed dose of Idebenone given to a wide range of patient ages and body weights prevented correlation of the observed effects with drug exposure. Further, the age difference between the treatment and placebo groups also complicated the interpretation of the study results. Larger randomized controlled studies are currently ongoing from the same research group to further investigate the potential therapeutic role of Idebenone in DMD.

#### 3.2.2. Polyphenolic Compounds

Polyphenolic compounds are an exciting new group of potential therapeutic candidates for DMD and have recently gained popularity due to their antioxidant, anti-inflammatory, cancer preventative, and cardiovascular benefits [146,147]. Of these compounds, Resveratrol, Quercetin and epigallocatechin-3-gallate (EGCG) are showing promising results as an effective treatment for muscular dystrophy.

#### 3.2.3. Resveratrol

Resveratrol (*3,5,4’-trihydroxystilbene*) is a polyphenolic extract of red wine that is also found in high concentrations in grape skins and the tree bark and root of certain tree species [148,149,150]. Resveratrol’s actions are thought to be primarily mediated through the *NAD* (*þ*)-dependent deaceytlase, Sirt1 [151,152]. Resveratrol increases the expression and activity of Sirt1 by molecules such as AMP-dependent protein kinase [153]. Sirt1 is a nucleocytoplasmic shuttling protein [154] and its translocation into the nucleus induces superoxide dismutase 2 (Sod2/Mn-Sod), decreases reactive oxygen species (ROS) levels, and inhibits oxidative stress-induced cell death [155]. Furthermore, both Resveratrol and Sirt1 activation lead to increased expression of peroxisome-proliferator-activated receptor-γ co-activator 1 (PGC-1α) [156]. PGC-1α regulates muscle fibre type determination, inducing a shift from fast to slow fibre type, mitochondrial biogenesis and oxidative capacity [157]. Another direct downstream target of PGC-1α is utrophin. Utrophin is a dystrophin homolog and is similar in size and structure to dystrophin [158]. Although it is primarily located at the neuromuscular junction in adult muscle, it can also functionally take the place of dystrophin throughout the muscle membrane. Thus the potential for resveratrol to potentiate therapeutic efficacy at both the metabolic and structural level of dystrophic skeletal muscle is obvious.

Research into the effects of resveratrol in the *mdx* mouse model of DMD has only been published within the past 5 years [157,159,160,161,162,163,164]. Hori *et al.* [162] supplemented 9 week old *mdx* mice with resveratrol (500 mg·kg^−1^·day^−1^) for 32 weeks and found that resveratrol reduced muscle wasting (less muscle wasting and non-muscle interstitial tissue deposition in the biceps) compared to *mdx* mice fed a control diet. Within the muscle, resveratrol reduced oxidative damage as shown by the immunostaining of nitrotyrosine and 8-hydroxy-2′-deoxyguanosine, and suppressed the up-regulation of NADPH oxidase subunits Nox4, Duox1, and p47phox. Resveratrol also reduced the number of α-smooth muscle actin (α-SMA) + myofibroblast cells and endomysial fibrosis in the biceps femoris. Even though resveratrol did not reduce the secretion of transforming growth factor-β1 (TGF-β1), a key regulator of collagen deposition and fibrosis, it significantly blocked TGF-β1 signal transduction. 

In addition, Selsby *et al.* [157] treated 4 week old *mdx* mice with resveratrol (0 mg·kg^−1^·day^−1^, 100 mg·kg^−1^·day^−1^ or 400 mg·kg^−1^·day^−1^ resveratrol) for 8 weeks. Animals fed a diet containing 100 mg·kg^−1^·day^−1^ resveratrol demonstrated increased specific force in the fast twitch EDL muscle and fatigue resistance in the slow twitch soleus muscle, however failed to increase resistance to injury in either muscle. Despite improved function, the authors did not investigate resveratrol’s effect on disease pathology. Furthermore, they also reported that resveratrol did not increase the protein expression of utrophin. Similar to Hori *et al.* [162], Selsby *et al.* [157] administered food orally and therefore dosage could only be estimated which may explain the reason for lack of increase in utrophin. It should be noted that that the dosage of 400 mg·kg^−1^ administered to the animals resulted in a number of deaths over the 8 week supplementation period indicating possible toxicity with this compound. However, since other investigations using this dosage or higher have failed to show this, it may indicate a source dependent effect rather than an effect of the resveratrol dose, per se [158].

To overcome the lack of dosage precision in previous studies, Gordon and colleagues [163] supplemented 5 week old *mdx* mice with resveratrol via oral gavage each day for 10 days. A range of dosages (0, 10, 100, or 500 mg·kg^−1^) were used initially to determine the optimal resveratrol dosage for Sirt1 activation prior to the beginning of an intervention trial. Similar to Selsby *et al.* [157], 100 mg·kg^−1^ was shown to be most effective, and thus used for subsequent experimental testing. *Mdx* results showed that 100 mg·kg^−1^ was the only dosage to significantly increase Sirt1 gene expression and thus further analysis was performed using this dosage. Compared to water treated control, Resveratrol treatment reduced immune cell infiltration by 21% (assessed via H&E stain) and 43% (assessed via CD45 immunohistochemistry—a more specific marker of inflammatory cell infiltration), reduced macrophage infiltration by 48% (assessed via F4/80 immunohistochemistry) and increased pro inflammatory cytokine IL-6, but not TNF-α. Finally, PGC-1α, and utrophin gene expression were increased by 27% and 43%, respectively. However, utrophin, Sirt1, and PGC-1α protein content did not change following 10 days supplementation. 

In a follow up study, Gordon *et al.* [164] recently determined if the beneficial effects observed in their previous study could be translated into improved muscle function, pathology and oxidative capacity in young mice. Four to five week old male *mdx* mice were randomized into control (water) or Resveratrol-treated groups (100 mg·kg^−1^) and dosed every other day for 8 weeks. Resveratrol mediated substantial improvements in rotarod performance (rotating rod with forced motor activity being applied) and in-situ peak tension by 53% and 17%, respectively. Slight improvements in centronucleated fibres and oxidative stress were also observed. Resveratrol did not affect total immune cell infiltrate at 12 weeks of age, and had no effect on oxidative capacity. Despite the small changes in muscle pathology, Resveratrol was capable of improving muscle function in *mdx* mice. The authors suggest that the likely mechanism is a Resveratrol-mediated reduction in immune cell infiltrate at the early stages of this disease, which is consistent with their previous findings [163].

Recently, Ljubicic and colleagues [160] demonstrated significantly higher Sirt1 activity and protein levels, as well as PGC-1α acetylation following 6 weeks of resveratrol supplementation. Using a similar dosage of 100 mg·kg^−1^·day^−1^ that has shown to be effective in previous studies, 12 weeks old *mdx* mice demonstrated evidence of fibre type transition towards slower, more oxidative fibres with concomitant increases in mitochondrial biogenesis and expression of slower myosin heavy chain isoforms. These changes were evoked independent of AMPK signaling-induction. In addition, the *mdx* mice were also supplemented with a higher dosage (500 mg·kg^−1^·day^−1^) for the same period, but this was less effective and supports previous findings of no added benefit at dosages higher than 100 mg·kg^−1^·day^−1^. 

Finally, protein lysine acetylation/deacetylation is emerging as an important regulatory mechanism of cellular functions. Transcriptional co-activator P300 acetylates histones and transcription (co-)factors and controls physiological processes—overexpression of P300 induces cardiomyocyte hypertrophy *in vitro* and *in vivo*, whereas down-regulating P300 ameliorates cardiomyopathy [165]. Resveratrol has been shown to down-regulate P300 protein levels and thus may improve the cardiac pathology characteristic of DMD. To explore this concept, 9 week old mice were fed Resveratrol (4 g·kg·meal^−1^) for 32 weeks [161]. At the age of 41 weeks, pathological increases in heart weight, the heart weight-to-body weight ratio, and left ventricular cardiomyocyte diameter were suppressed in Resveratrol-treated *mdx* mice when compared to untreated *mdx* and control C57BL/10 mice. Furthermore, elevations of atrial natriuretic peptide (ANP) mRNA, a marker of cardiac hypertrophy, in the *mdx* heart was also suppressed by resveratrol treatment. Resveratrol suppressed the increase in myocardial P300 protein level normally seen in *mdx* mice, but had no effect on Sirt1 protein and mRNA levels in the heart. Resveratrol also increased histone deacetylase activity and/or inhibited histone acetyltransferase activity. Although results are promising, more research is needed to determine if Resveratrol slows or reverses the progression of cardiac dysfunction administered even after the onset of cardiomyopathy in the dystrophin-deficient heart.

#### 3.2.4. Quercetin

Another natural polyphenolic flavonoid that is a potent activator of Sirt1 is Quercetin. Quercetin can demonstrably induce deacetylation and activation of PGC-1α when taken orally, which stimulates mitochondrial biogenesis in skeletal muscle [161]. In addition, Quercetin, is an antioxidant and displays anti-inflammatory characteristics in a variety of cells [161]. Both improved mitochondrial function and reduced ROS-mediated inflammation via Quercetin supplementation may provide benefit to dystrophic skeletal muscle. Only two studies to date have published the effects of Quercetin supplementation on dystrophic muscle—both were performed using the *mdx* mouse model of the disease and by the same laboratory group. Hollinger *et al.* [166] supplemented 3 months old *mdx* mice with a 0.2% Quercetin-enriched diet for 6 months and reported various beneficial effects in the diaphragm compared to unsupplemented controls. Of note, there was a significant reduction in muscle histopathology whereby muscle fibre number was preserved while inflammation and fibrotic infiltration was reduced. These improvements occurred alongside increased expression of various oxidative genes including the mitochondria transcription factor *Tfam* and the mitochondrial encoded transcript *CoxII*, suggesting that increasing the capacity of the mitochondria to produce ATP is key to reducing the rate of skeletal muscle degeneration in dystrophin-deficient muscle. However, it was unclear as to whether Quercetin was mediating its effects via a PGC1-α-dependent pathway as the transcription of downstream targets was comparable to controls, as was utrophin expression which has been shown to be positively regulated by PGC1-α in gene transfer studies.

In hearts from the same animals [166] and an additional set of 3 weeks old *mdx* mice that were supplemented with 0.2% dietary Quercetin until 6 months of age, Ballmann *et al.* [167] have demonstrated similar efficacy in attenuating cardiomyopathy. Used as a model in which to explore the “preventative” efficacy of Quercetin, young *mdx* mice showed a reduced propensity for cardiopathology as detected by a 50% reduction in matrix metalloproteinase 9 expression. Increased mitochondrial density (as inferred by a higher cytochrome c content), utrophin and SOD expression was also detected in this “preventative” model compared to unsupplemented controls. This suggests that the protective effects exerted by Quercetin are due to PGC1α-mediated expansion of the mitochondrial pool and a greater propensity for ROS buffering. In the “rescue” model of older *mdx* mice, Quercetin supplementation attenuated ventricular damage and prevented cardiac hypertrophy compared to unsupplemented controls. This seemed to be related to lower TGF-β1 expression and not due to a PGC1α-induced response as per the “preventative” model.

While the potential efficacy of quercetin is yet to be investigated in human DMD patients, these studies in the *mdx* mouse model show promise and warrant further investigation regarding potential physiological outcomes and the precise mechanisms via which polyphenols apparently allay skeletal muscle and cardiac pathology associated with dystrophin deficiency.

#### 3.2.5. Epigallocatechin Gallate (EGCG)

Consumed all over the world, green tea is recognised for its antioxidant and cancer chemoprevative properties [168]. Made from unfermented leaves, green tea is rich in catechins polyphenols of which epigallocatechin gallate (EGCG) is the most abundant (30%–50% of total polyphenols) [169]. Green tea polyphenols (GTP) and its major active constituent EGCG trap hydroxyl and peroxyl radicals and have shown to be superior ROS scavengers compared to vitamins C and E [170,171,172]. In addition, EGCG may also improve mitochondrial dysfunction with evidence showing rescued mitochondrial complex I and ATP synthase catalytic activities and restored oxidative phosphorylation efficiency in cultured lymphoblasts and fibroblasts from Down syndrome patients [173]. Furthermore, EGCG has been shown to increase Sirt1-dependent PGC-1α deacetylation, *nuclear respiratory factor 1* and mitochondrial transcription factor A protein levels, and mitochondrial DNA content, concomitant with mitochondrial biogenesis [173]. Although the exact mechanism of action of GTP and EGCG are not known, current evidence suggests that the benefits seem to be related to their high antioxidative capacity [171].

Several groups over the past 10 years have investigated the therapeutic effects of GTP mixtures and EGCG alone via subcutaneous (sc) or oral administration in *mdx* mice. One Swiss group in particular (Dr Ruegg’s laboratory), have published a number of papers in this area. In 2002, they showed that a diet containing 0.01% or 0.05% GTP administered to *mdx* mice from birth (through their mother before weaning) significantly reduced necrosis in fast-twitch, but not slow-twitch fibres [174]. In a follow up study, the investigators used a higher dose of GTP (0.25% wt/wt) compared to the previous study, and also tested the effectiveness of EGCG alone at a dose corresponding to the EGCG content in the 0.25% GTP group [175]. The mixtures were given via the diet to 3 weeks old dystrophic mice, either for 1 week to determine if the mixtures could offset the large muscle degeneration that normally occurs at 3 to 4 weeks of age; or for 5 weeks, to investigate the effects over the proceeding regeneration period as well (usually completed by 8 weeks of age). Overall, EGCG was slightly more efficacious compared with GTP, with both substances delaying muscle necrosis and protecting the EDL muscle but not the soleus muscle (confirming their previous findings of preferential fibre type effects). The investigators also observed improvements in structural and contractile properties of the dystrophic muscle, notably force output and resistance to fatigue [175]. The authors concluded that GTP and EGCG protect muscle cells by scavenging ROS and by improving the antioxidant (glutathione) balance and reducing oxidative stress [174,176].

In a recent study, Nikae *et al.* [177] compared administration routes and dosages of EGCG to determine the most effective for reducing the onset of dystrophic injuries [177]. Over a 5 weeks period, two groups of 3 weeks old *mdx* mice were injected sc with either saline or a daily average of 3 or 6 mg/kg EGCG, or fed with either a diet containing 0.1% EGCG (equivalent to 180 mg/kg per day) or a control diet [177]. At a dosage of 180 mg·kg^−1^·day^−1^, EGCG administered through the diet was found to be most effective with significantly better improvements in serum muscle-derived CK, a marker of muscle damage; isometric force; oxidative stress and fibrosis tissue. No obvious signs of toxicity were evident. It should be noted, however, that improvements were slightly less than those observed previously for sc injection when administered immediately after birth [177].

Other studies have confirmed the findings of Ruegg’s laboratory. Subcutaneous injection of 5 mg·kg^−1^ EGCG 4 times per week from birth for 8 weeks, has been shown to reduce the phenotypic onset of muscular dystrophy in *mdx* mice as evidenced by near normal levels of muscle-derived CK levels, reduced markers of aging and “wear-and-tear” as indicated by a significant reduction in fluorescent lipofuscin granules per unit volume of soleus and diaphragm muscles, less fibrosis and necrotic myofibres, an increased number of normal myofibres, and enhanced expression of utrophin, a homologue of dystrophin [178]. A diet containing 0.5% GTP given to *mdx* mice from gestation until 6 weeks after birth increased voluntary wheel running activity and citrate synthase activity (marker of oxidative capacity) in gastrocnemius muscle, lowered lipid peroxidation (an index of membrane degradation) in cardiac and gastrocnemius muscles, and decreased sarcolemmal damage [179]. In another study from the same lab, the histopathology of *mdx* tibialis anterior muscles was improved and NF-kB (inflammatory pathway) in the nuclei of the regenerating myofibres was down regulated using a similar dosage [180].

The results from the aforementioned studies are very promising and suggest that EGCG may be of benefit for the treatment of DMD patients. In 2010, the first human trial was started with investigators wanting to examine safety and tolerance of 10 mg·kg^−1^ EGCG for 12 weeks, and efficacy up to 36 weeks in patients with DMD. The multi-centre, prospective, double blind, placebo controlled, randomized pilot study is coordinated by Dr. Friedemann of Charite University, Germany, and is due for completion in 2018.

### 3.3. Adenine Nucleotide Salvage and *de Novo* Synthesis Promoters

The maintenance of ATP levels in skeletal muscle is influenced by the balance between degradation and re-synthesis of the adenine nucleotide pool which is in a constant state of flux. The energy status of the muscle and the capacity of the metabolic pathways of purine *de novo* synthesis and purine salvage in the muscle are primary determinants. In healthy resting muscle, purine salvage ensures that the majority of the purine nucleotide pool is recovered within the muscle and not lost via efflux of metabolites into the blood. The enzyme responsible for purine salvage is Hypoxanthine Guanosine Phosphoribosyl Transferase (HGPRT), which is critical for good health as demonstrated by the debilitating nature of its absence in Lesch Nyhan Syndrome [181]. During skeletal muscle metabolic stress and/or catabolism, purine nucleotides can be rapidly degraded to inosine monophosphate (IMP) and hypoxanthine [182], and, following normal recovery conditions, up to 90% of the produced hypoxanthine is capably salvaged via the purine nucleotide salvage pathway [183]. In this pathway, hypoxanthine is recovered to IMP by HGPRT before being converted to adenylosuccinate by adenylosuccinate synthetase, and subsequently to AMP by adenylosuccinase. Effective mitochondrial oxidative phosphorylation and sufficient levels of both ADP and AMP are required for complete repletion of ATP by purine salvage. However, in persistent metabolic stress and catabolic states such as that evident following intense periods of exercise [182] and in conditions of pathological wasting associated with DMD [8,28], ATP demand is persistent and compounds purine base (inosine and hypoxanthine) production and loss from skeletal muscle (which are subsequently eliminated as uric acid in urine) never to be reclaimed. In this instance, ATP repletion would be achieved via *de novo* ATP synthesis via the pentose phosphate pathway (PPP) in which glucose-6-phosphate is converted to phosphoribosyl-1-pyrophosphate and IMP via a series of reactions. This is a rate limiting step in the formation of ATP [184] and has been shown to be improved with ribose feeding [185].

There are fiber type differences in both the degradation and re-synthesis capacities of adenine nucleotides. Karatzeferis *et al.* [186] demonstrated that Type II fibres exhibit almost complete degradation of ATP following a maximal sprint whilst in Type I fibres ATP degradation is non-existent. A similar effect is observed in rat skeletal muscle where fast-twitch muscle exhibits greater degradation rates than that of slow-twitch muscle [187]. While the precise metabolic pathways responsible for the recovery of purines in different muscle fibre types have not been established in human muscle, the capacity for *de novo* ATP synthesis is demonstrably slowest in fast-twitch muscle and fastest in slow-twitch red fibres from the rat [187]. This data highlights that fast-twitch fibres are at a disadvantage during chronic metabolic stress and therefore more likely candidates for autologous cell death induction due to the mismatch between purine loss and *de novo* ATP recovery. This scenario provides a potential explanation for Type II fibres being preferentially degraded in DMD muscle [188], and presents an avenue for targeted supplement manipulation of repair and recovery. 

At the height of considering DMD as a predominantly metabolic disorder, trial treatments were aimed at increasing the total adenine nucleotide (TAN) availability by: (1) preventing the loss of valuable adenine nucleotides; and (2) increasing the supply of metabolites and efficacy of metabolic pathways and associated support systems. One such trial delivered a cocktail of nucleosides and nucleotides (adenosine, AMP, ADP, ATP, guanosine, guanosine monophosphate, uridine, uridine monophosphate and cytidine monophosphate) to 11 DMD boys which showed some improvement in enzymatic and functional capacity [189]. However, more targeted therapies were approached following this, which will be discussed hereafter.

#### 3.3.1. Adenylosuccinic Acid

A seminal study that illustrates the critical role of ATP depletion in dystrophinopathy comprised a 10 year clinical trial of adenylosuccinic acid (ASA) treatment [190]. ASA stimulates the PNC producing fumarate to fuel TCA cycling and adenine nucleotides that can increase ADP re-synthesis and availability to mitochondrial ATP production. Increasing dosage of daily ASA treatment (25 mg·kg^−1^·day^−1^ to 600 mg·kg^−1^·day^−1^) induced vast improvements in a DMD patient who commenced supplementation from 2.5 years of age. Instantaneous increases in energy, endurance and stamina were observed following commencement of ASA supplementation with significant maintenance of muscle strength and function (*i.e.*, able to stand erect, walk without falling and rise from the floor) noted over the supplementation period. In addition, ASA induced a four-fold reduction in serum CK levels indicative of reduced muscle damage, and improved the typical DMD histopathological hallmarks including the ratio of regeneration compared to necrosis [190]. Such improvements in the dystrophic condition readily subsided upon discontinuation of ASA supplementation highlighting that ongoing support of the mitochondria is pivotal to mitigating disease progression.

A prominent feature of a muscle biopsy taken from the DMD patient some 4.5 years following commencement of ASA supplementation was a lack of fatty tissue infiltration. A prominent feature of dystrophic disease progression is accumulation of fatty tissue [191,192,193] which may be reflective of a higher propensity for dystrophic muscle to produce lipids. Culturing of healthy and DMD human myocytes revealed that in the presence of decreasing concentrations of foetal bovine serum, lipid production became negligible in healthy cultures while DMD cultures continued to produce lipid droplets irrespective of serum concentration [193]. The enhanced ability to produce lipid droplets in DMD myocytes was pinpointed to a dysfunction at the level of isocitrate dehydrogenase in the TCA cycle—this dysfunction was reportedly eliminated following the addition 1.2 × 10^−6^ M ASA [194]. ASA’s ability to improve multiple facets of the dystrophic phenotype appears to lie in its capacity to support both the TCA cycle and the adenine nucleotide pool. These studies importantly highlight the necessity for further investigation into the ways in which metabolic capacity can be enhanced by ASA to improve the phenotypic progression of DMD and the quality of life of patients.

#### 3.3.2. Ribose

The pentose monosaccharide, ribose, has been used ostensibly to increase nucleotide recovery via the purine salvage and *de novo* synthesis pathways with the ultimate goal of enhancing depressed cellular ATP levels following exercise [185,195], and in cardiac [196] and various metabolic [195] diseases. Supplemental ribose acts as an alternative substrate for *de novo* ATP synthesis by entering the PPP where it is rapidly converted to ribose-5-phosphate (R-5-P) by ribokinase. R-5-P is subsequently catalyzed to phosphoribosyl-1-pyrophosphate (PRPP) to increase IMP availability to the purine salvage pathway. As such, the dependency of *de novo* ATP synthesis on glucose-6-phosphate availability and on the rate-limiting function of glucose-6-phsophate dehydrogenase (G-6-PDH) is eliminated.

While beneficial effects have been observed in healthy individuals recovering from sprint training regimes [185] as well as patients with myoadenylate deaminase (MAD) deficiency and McArdles disease (glycogen phosphorylase deficiency) following dietary ribose supplementation, only one study to date has been performed in DMD patients. Griffiths *et al.* (1985) supplemented 5 DMD boys aged 6.5 to 11 years with 2 × 250 mg ribose dosages per day over 12 weeks which had no effect on total muscle phosphorous (including ATP, PCr, Pi, PDi and PCr + Pi) as measured by ^31^P-NMR, plasma CK levels, muscle strength, or functional measures [197]. However, in this study, only differences in PCr and Pi were observed between the muscle metabolites of DMD compared to healthy patients prior to treatment. Importantly, ATP content was shown to be comparable between DMD and healthy patients and this may be a better reflection of the improved chronic status of the muscle relative to the acute nature of PCr metabolism—other studies since have demonstrated up to 50% reductions in resting ATP levels in DMD skeletal muscle [8]. A notable criticism of this study was that it used a relatively minor dosage of 250 mg twice daily. Studies is MAD deficient patients demonstrated that low dose ribose supplementation of 450 mg was ineffective at reducing exercise-associated soreness and cramping whereas a supplementation rate of 4 g·dose^−1^ was proven beneficial [198,199]. Thus the use of moderate to high dose ribose supplementation warrants further investigation in DMD.

#### 3.3.3. Allopurinol

In an effort to limit the loss of adenine nucleotides from skeletal muscle as hypoxanthine, supplementation with allopurinol has been extensively investigated. Allopurinol works via its metabolite oxipurinol which inhibits the activity of xanthine oxidase in vascular endothelium [200]. This moderates the conversion of purines to uric acid to maintain relatively high plasma hypoxanthine concentrations relative to the muscle, thereby theoretically reducing the flux of purines from skeletal muscle. This would afford beneficial effects to dystrophic skeletal muscle by reducing the need for purine repletion via *de novo* synthesis and promoting the re-circulation of hypoxanthine into ATP-synthesis pathways [201]. As there is increased uric acid excretion (and therefore a need for purine salvage) and a requirement for energy support in DMD patients, supplementation with allopurinol poses major benefits.

During a 6–12 week trial of 100 mg allopurinol, significant improvements in manual function was observed which was maintained for a subsequent 6 months [201]. Biochemical studies have demonstrated similar benefits—following body weight-corrected supplementation of DMD patients, improvement in levels of metabolites that can stimulate ATP resynthesis was observed [202] with improvements in the concentrations of ATP [203,204], ADP, GTP, guanosine diphosphate, IMP, adenylosuccinate, adenine, hypoxanthine and guanine noted following a trial with allopurinol based on age [203]. This improvement in energy potential was partnered with an increase in the charge of dystrophic mitochondria from 51% in the placebo group to 83% in allopurinol-treated patients respectively [203]. This data suggests that the capacity for ATP production is significantly improved by inhibiting purine catabolism.

In the quadriceps, biceps and adductor muscles, allopurinol supplementation of 10 mg·kg^−1^·day^−^^1^ showed either stabilisation or improvement in strength in the DMD patients in the earliest phases of the disease [205]. As expected, decreased uric acid excretion was observed even in the later stages of the disease [60]. However, Kulakowski *et al.* [206] and Tamari *et al.* [207] only noted functional and biochemical improvement in some patients (generally in the earlier stage of the disease) following 6 months of therapy [206,207], highlighting that allopurinol therapy might not afford the widespread benefit that was originally touted.

Numerous researchers have subsequently failed to replicate the beneficial effects of allopurinol described in previous experiments as an effective treatment for DMD. One of the first to reject the findings of Thomson and Smith [204] was Bakouche *et al.* [208] who gave a variety of muscular dystrophy patients (including DMD) 100 mg of allopurinol daily for a month and 300 mg daily for a subsequent two months and observed no overall improvement [208]. It should be noted, however, that the age of patients utilized in this study was in excess of 20 years, which is a significantly older cohort compared to any other study that had previously investigated allopurinol. Given the progressive nature of DMD and the relative lifespan of sufferers, it is likely that no treatment would be beneficial at this stage of disease progression.

In ambulatory dystrophic males from 6 to 12 years of age, no significant improvement in functional muscle tests or serum CK was observed following supplementation with 100 mg allopurinol daily for one year [209]. To increase sample size, all 6 patients received allopurinol for a further 8 weeks to no avail, therefore the authors concluded allopurinol to have no therapeutic effect [209]. Similarly, following an 8 months 150 mg allopurinol supplementation in dystrophic boys aged 5 to 13, no clinical improvement, but rather, deterioration was observed in functional and strength tests [210] with comparable findings observed following two 3-month trials of 100 mg allopurinol [211].

In another trial that supplemented allopurinol to 25 DMD boys aged 5–18 years based on weight (no dose exceeding 150 mg daily) for 12 months, no clinical improvement in muscle and pulmonary function tests and serum CK and PK levels were observed by the authors despite a decrease in blood uric acid levels [212]. Furthermore, following daily 200–300 mg doses of allopurinol to 5 DMD boys aged 6–13 years for 6 weeks [197] and 300 mg daily to 14 DMD boys aged 7–16 years for 18 months [213], no improvement in phosphate metabolites (including ATP levels), functional measurements and strength were observed again despite decreased excretion of uric acid. Overall, these studies report comparable findings using similar assessment techniques and were reported in quick succession indicating that allopurinol therapy may not be a useful adjunct therapy for the treatment of DMD.

The majority of studies that have deemed allopurinol therapy to be ineffective for DMD treatment noted a deterioration of muscle function during and following the supplementation period [197,210,211,212,213]. This may be reflective of the age of participants in these trials and to what degree the disease had progressed, with the baseline measurements of these studies indicating that younger patients were able to perform better on functional tests and generally saw some sort of improvement from allopurinol treatment. Even when young patients are included in clinical trials, often there is a relatively small age range used (*i.e.*, 6–13 years) that spans a broad range of disease phenotype within an experimental group (*i.e.*, a 6 years old might still be in a preclinical stage while a 13 year old may have lost ambulatory capacity). It is well-known that as DMD progresses, muscle wastage intensifies with the culmination of fibrotic, non-functional muscle and strength declines [214]. Therefore, the inclusion of DMD patients of older age and who are non-ambulatory (or thereabouts) limits the potential of such metabolic therapies as there is a significant reduction in treatable muscle mass. This may explain the conflicting findings and highlights the need for clinical trials to implement early intervention strategies when considering metabolically-targeted therapies and their ability to maintain long term efficacy.

## 4. Summary and Recommendations

**Figure 3:**
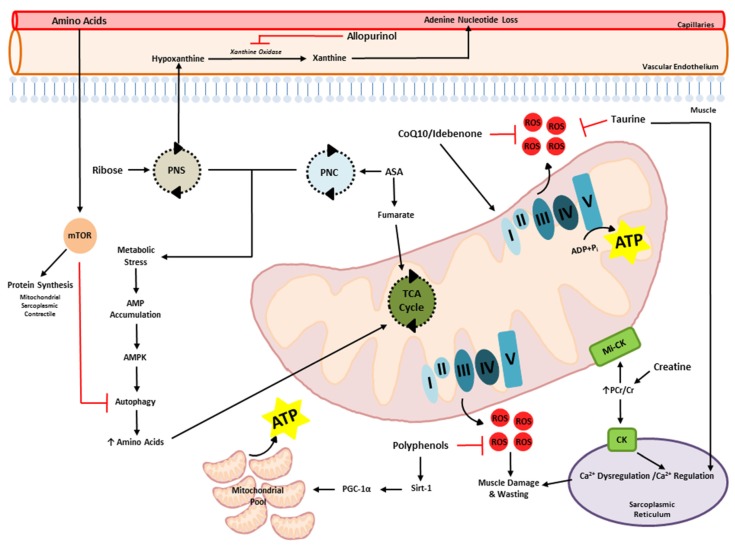
Metabogenic and nutriceutical supplements to promote bioenergetical potential and moderate muscle wasting in DMD. Dystrophin-deficient muscle is characterised by the inability to produce sufficient ATP to buffer increased (Ca^2+^), limit degeneration and to fuel regeneration. Addition of ATP precursors (amino acids, ASA, creatine and ribose) would expand the potential energy pool and subsequently increase ATP production through stimulation of the various metabolic pathways. An additional benefit of AAs would be to augment muscle protein synthesis and inhibit macroautophagy via direct stimulation of mTOR. Limiting the loss of adenine nucleotides via allopurinol would further expand the potential energy pool and mitigate the need for energy-consuming *de novo* ATP synthesis. Buffering of ROS-mediated (CoQ10/Idebenone, polyphenols and taurine) and Ca^2+^-induced muscle damage (creatine and taurine) and stimulation of mitochondrial biogenesis (polyphenols) would limit energy expenditure on regeneration and augment bioenergetical expansion of dystrophic skeletal muscle. Figure abbreviations: ADP—adenosine diphosphate; AMP—adenosine monophoshate; AMPK—adenosine monophophate activated protein kinase; ASA—adenylosuccinic acid; ATP—adenosinie triphosphate; Ca^2+^—calcium; CK—creatine kinase; Cr—creatine; Mi-CK—mitochondrial creatine kinase; mTOR—mammalian target of rapamycin; PCr—phosphocreatine; PGC-1α—peroxisome proliferator-activated receptor gamma coactivator 1 alpha; P_i_—inorganic phosphate; PNC—purine nucleotide cycle; PNS—purine nucleotide salvage pathway; ROS—reactive oxygen species; TCA—tricarboxylic acid.

Herein, we have reviewed pre-clinical and clinical experimental data derived from both animal models of, and human patients with, DMD, using numerous metabogenic and nutriceutical compounds in an attempt to mitigate dystrophinopathy. Through a variety of different intracellular mechanisms (as summarised in Figure 3), many of these compounds improve various indices of DMD skeletal muscle pathology. As the majority of the compounds that we have reviewed can be purchased from nutriceutical suppliers, and therefore readily implemented by parents and health care providers into established treatment regimens, we thought it was pertinent to categorise the reviewed compounds according to their level of established therapeutic efficacy and summarise the beneficial effects of each alongside the most efficacious dosage or dosage range (Table 1). It is our opinion, that the biggest limiting factor to families and health care providers wanting to make informed decisions about the suitability and feasibility of implementing such compounds into treatment regimens, is that there is few efficacy data derived from long term supplementation/treatment strategies (*i.e.*, 10 years+), As such, it is difficult to identify the effect these compounds have on the long term clinical progression of DMD. Maintaining realistic expectation regarding the degree of therapeutic efficacy metabogenic compounds can elicit is therefore, recommended. For example, it would be unrealistic to presume that the commencement of metabogenic therapy in advanced stage DMD could reverse the loss of ambulation or cardiomyopathy. In contrast, the implementation of treatment during the pre-clinical stages could act as a disease modifier to significantly alter the phenotypic course of the disease, thus having very obvious impacts on patient quality of life.

**Table 1:** Categorised summary of metabogenic compound efficacy and dosage for the adjunct treatment of DMD, based upon available literature.

Category	Compound	Dosage Range	Summary of Beneficial Effects	Other
Apparently effective/safe to consume (in human DMD patients)	Creatine	3–5 g·day^−1^	Improved strength, maintenance of strength, increased lean muscle mass, attenuating exercise-induced fatigue, increased intramuscular energy stores	
	CoenzymeQ10/Idebenone	>400 mg·day^−1^	Decreased plasma CK levels, Improved respiratory functional measures	In conjunction with corticosteroid therapy: improved muscle strength,
	Adenylosuccinic acid	25–600 mg·kg^−1^·day^−1^	Increased energy, endurance and stamina, maintenance of muscle strength and function, reduced serum CK, reduced muscle necrosis and enhanced regeneration	Importantly ASA therapy was administered during preclinical DMD (2.5 years) and was well tolerated for 10 years.
Possibly effective	Glutamine	0.5–0.8 g·kg^−1^·day^−1^	Increased muscle protein synthesis rates following short-term treatment	No effect on functional assessment tests, body composition/lean muscle mass, muscle protein breakdown following long-term treatment
	Resveratrol	100 mg·kg^−1^·day^−1^ in drinking water (mice)	Possibly, increased mitochondrial biogenesis, decreased inflammation, small reductions in oxidative stress and muscle fibre damage	Over- or under-dosing limits efficacy
	Quercetin	0.2% of diet (mice)	Possibly, reduced muscle degeneration, inflammation and fibrosis, attenuation of cardiomyopathy	
	Epigallocatechin gallate	Current clinical trial is establishing efficacy and safety at 10 mg·kg^−1^·day^−1^; Animal data suggests 180 mg·kg^−1^·day^−1^ equivalent human dose induces best benefits, albeit safety not established at this concentration	Possibly: Reduced serum CK levels, protection against muscle degeneration and fibrosis in fast-twitch muscle, reduced oxidative stress, reduced inflammation, increased force production and fatigue resistance	
Too early to tell/unclear	Taurine	Not established	Possible benefits include reduced oxidative stress/ROS damage, increased muscle contraction force and strength	
	l-arginine		Possibly, induction of slow fibre type transitions & utrophin expression (protective against damage)	
	Whey protein isolate	Not established	Possibly, induction of mitochondrial biogenesis	Currently on AUS clinical trial registry for efficacy evaluation with and without co-creatine supplementation
	Allopurinol	10 mg·kg^−1^·day^−1^	Improved skeletal muscle energy status, statbilisation or improvement of muscle strength	Several other trials have found no effect, Allopurinol might be most efficacious when combined with other metabogenic compounds
Not effective/not safe	Ribose	500 mg·day^−1^	None observed	In other metabolic diseases low dose therapy (500 mg·day^−1^) is ineffective, but efficacy is observed at a dosage of 8 g·day^−1^

## 5. Conclusions

Decades of clinical data suggest the therapeutic value of metabogenic nutriceutical supplements to promote energy and protein homeostasis in dystrophin-deficient muscle of DMD patients and animals models of the disease. Although the compounds described throughout this review were used ostensibly due to their metabogenic potential, it is interesting that very few studies have actually quantified metabolic changes and the downstream effects on skeletal muscle mass. Thus, while further work occurs to completely ascertain their efficacy as adjuvant treatments for DMD, these experiments should endeavor to establish whether metabolic augmentation is a component of any efficacy. Future experiments should also adopt standardized approaches to accurately compare efficacy between compounds and compound combinations. Whilst on a molecular level, Ca^2+^ handling dysregulation and accumulation secondary to the absence of dystrophin is central to the complex pathophysiology of DMD, it seems that the severe reductions in intracellular energy and AA status may be the immediate precursors to muscle necrosis, apoptosis and autophagic cell death pathways. This aspect warrants further attention from a treatment perspective, particularly given that experimental interventions driven at correcting the genetic cause of the disease are yet to be applicable to mainstay therapeutics despite several decades of consistent research. We have described the value of various metabogenic and nutriceutical supplements to adjuvate the treatment of DMD patients. While promising data has been derived using treatment regimens focused on isolated supplements, we suggest that greater therapeutic value could be gained from administering combined adjuvants in supplement regimens designed to target the various deficits and abnormalities evident in the metabolic milieu that regulates skeletal muscle energy balance and the maintenance of functional muscle mass.
